# Supplementary material for: The relationship between event boundary strength and pattern shifts across the cortical hierarchy during naturalistic movie-viewing
Source: bioRxiv. 2024 Jun 15:2024.04.10.588931. Originally published 2024 Apr 14. Preprint. [Version 2] doi: 10.1101/2024.04.10.588931 (PMC11030401; doi:10.1101/2024.04.10.588931)
Supplement: 1 [file NIHPP2024.04.10.588931V2-supplement-1.pdf]

bioRxiv preprint doi: https://doi.org/10.1101/2024.04.10.588931; this version posted June 15, 2024. The copyright holder for this preprint (which was not certified by peer review) is the author/funder, who has granted bioRxiv a license to display the preprint in perpetuity. It is made available under aCC-BY 4.0 International license.

SUPPLEMENTARY INFORMATION

	Proportion of human observers	
	**Minimum**	**Maximum**
Weak	.11	.19
Moderate	.20	.40
Strong	.40	.98

Supplementary Table 1. Agreement range for each event boundary strength category.

	Proportion of human observers	
	**Minimum**	**Maximum**
Weak	.12	.19
Moderate	.20	.38
Strong	.40	.98

Supplementary Table 2. Agreement range for each event boundary strength category after excluding pairs of event boundaries less than 6 seconds apart.

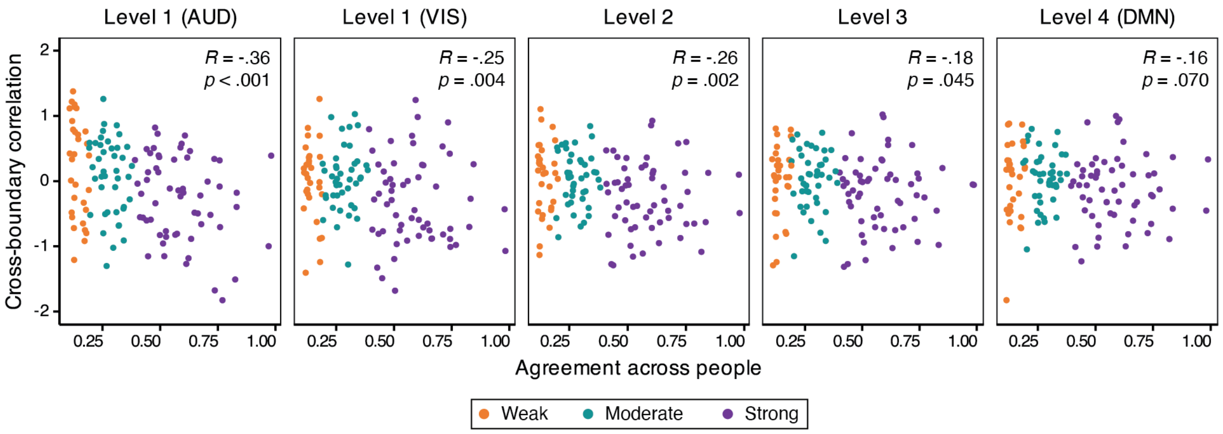

Supplementary Figure 1. Spearman's rank correlation between cortical pattern shift and across-observer boundary agreement at each cortical hierarchical level. Note that correlation time series was normalized (see Methods).

bioRxiv preprint doi: https://doi.org/10.1101/2024.04.10.588931; this version posted June 15, 2024. The copyright holder for this preprint (which was not certified by peer review) is the author/funder, who has granted bioRxiv a license to display the preprint in perpetuity. It is made available under aCC-BY 4.0 International license.

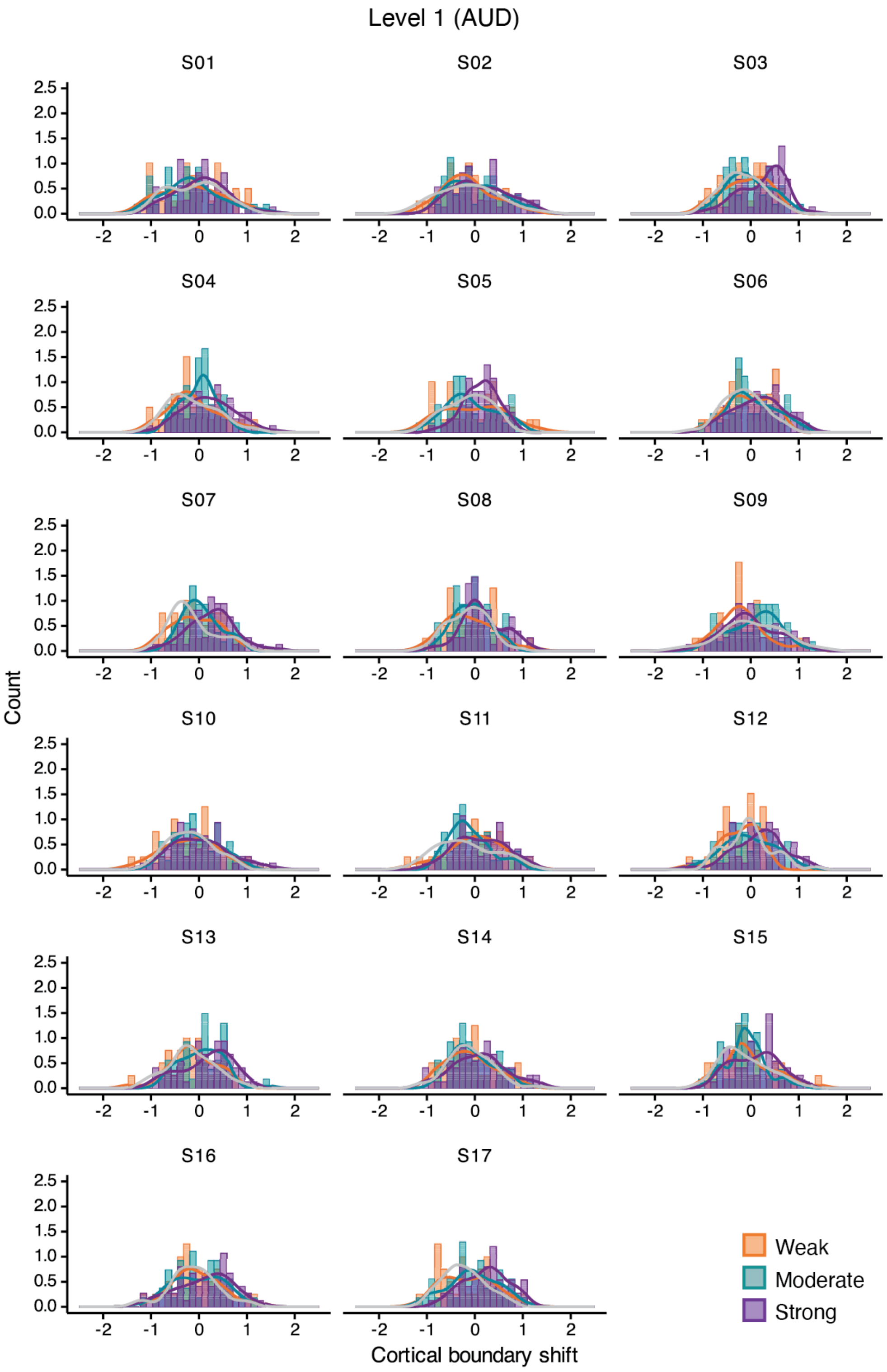

Supplementary Figure 2. Individuals' distributions of cortical boundary shifts for each boundary strength category in auditory processing areas (AUD) at level 1 of the cortical hierarchy. The gray line depicts a density curve of the non-boundary data.

bioRxiv preprint doi: https://doi.org/10.1101/2024.04.10.588931; this version posted June 15, 2024. The copyright holder for this preprint (which was not certified by peer review) is the author/funder, who has granted bioRxiv a license to display the preprint in perpetuity. It is made available under aCC-BY 4.0 International license.

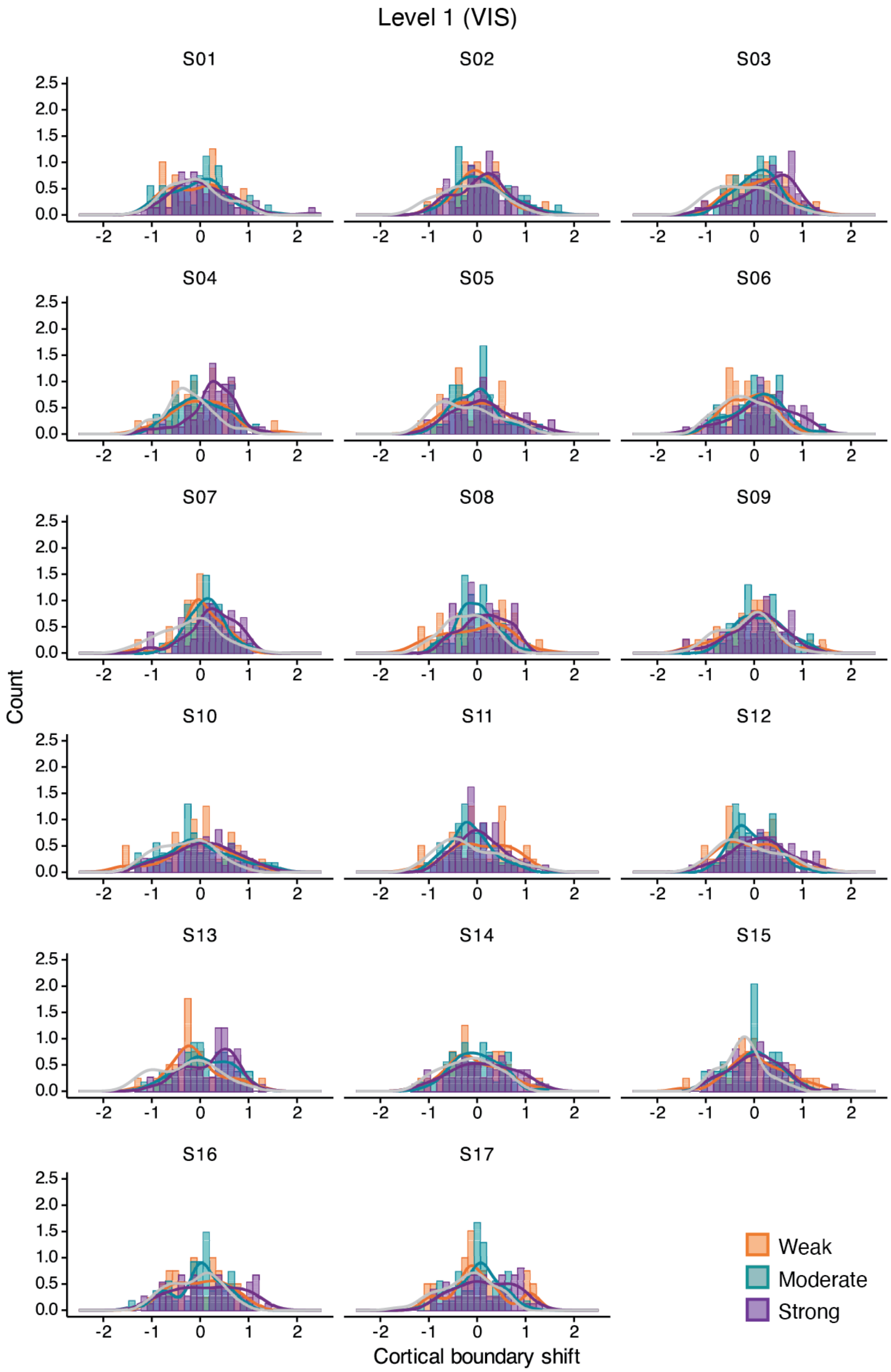

Supplementary Figure 3. Individuals' distributions of cortical boundary shifts for each boundary strength category in visual processing areas (VIS) at level 1 of the cortical hierarchy. The gray line depicts a density curve of the non-boundary data.

bioRxiv preprint doi: https://doi.org/10.1101/2024.04.10.588931; this version posted June 15, 2024. The copyright holder for this preprint (which was not certified by peer review) is the author/funder, who has granted bioRxiv a license to display the preprint in perpetuity. It is made available under aCC-BY 4.0 International license.

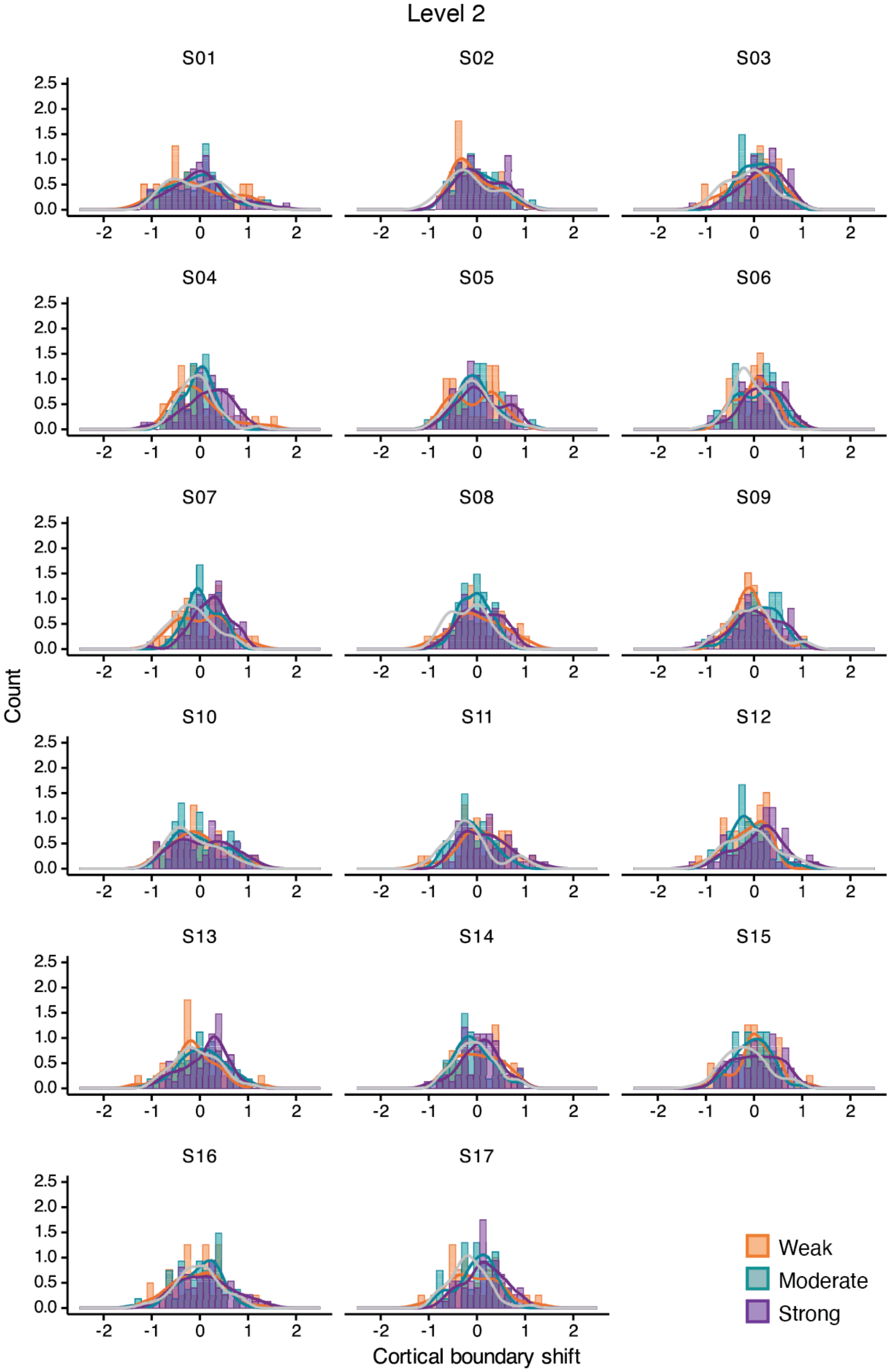

Supplementary Figure 4. Individuals' distributions of cortical boundary shifts for each boundary strength category in the areas at level 2 of the cortical hierarchy. The gray line depicts a density curve of the non-boundary data.

bioRxiv preprint doi: https://doi.org/10.1101/2024.04.10.588931; this version posted June 15, 2024. The copyright holder for this preprint (which was not certified by peer review) is the author/funder, who has granted bioRxiv a license to display the preprint in perpetuity. It is made available under aCC-BY 4.0 International license.

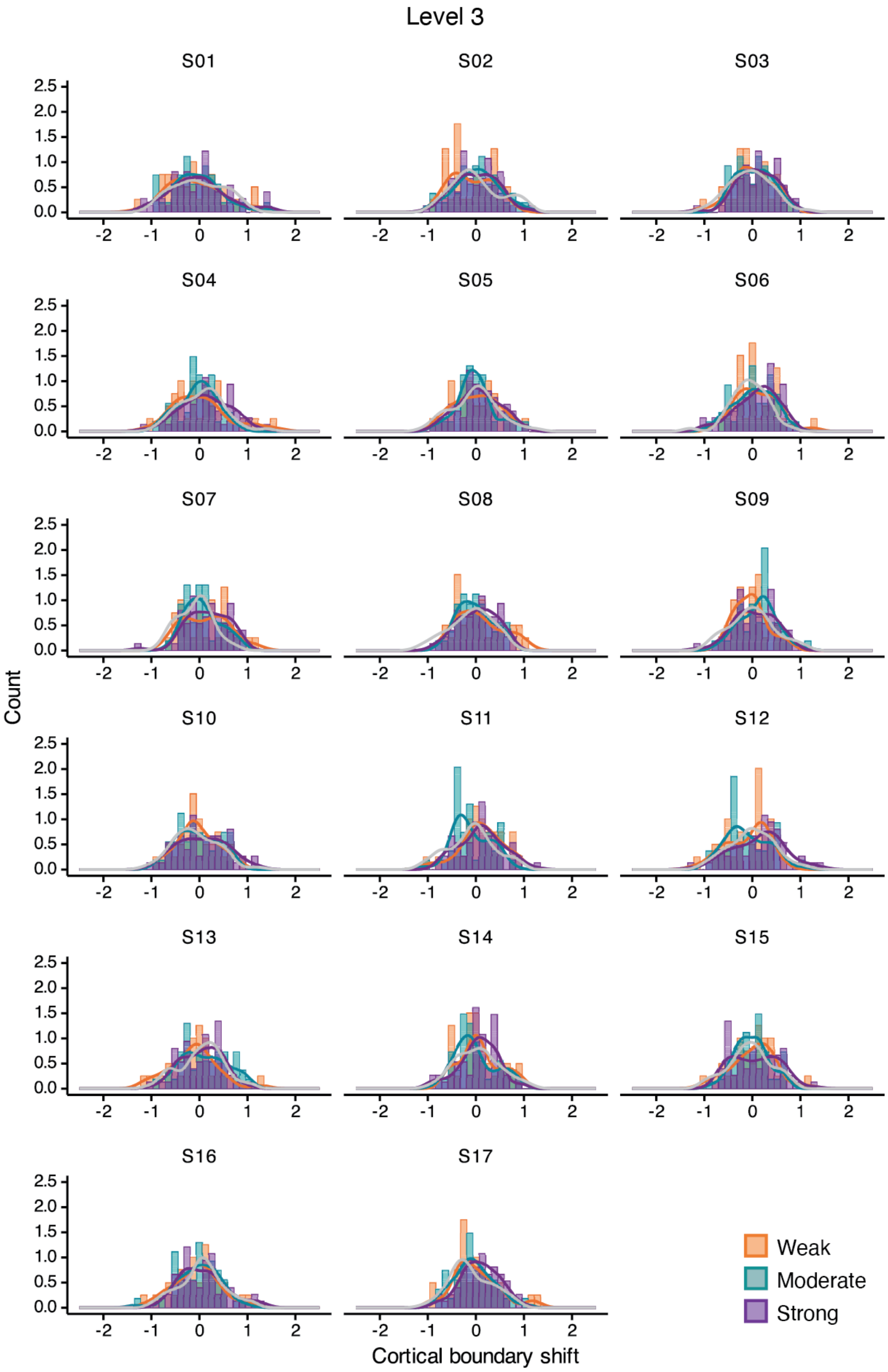

Supplementary Figure 5. Individuals' distributions of cortical boundary shifts for each boundary strength category in the areas at level 3 of the cortical hierarchy. The gray line depicts a density curve of the non-boundary data.

bioRxiv preprint doi: https://doi.org/10.1101/2024.04.10.588931; this version posted June 15, 2024. The copyright holder for this preprint (which was not certified by peer review) is the author/funder, who has granted bioRxiv a license to display the preprint in perpetuity. It is made available under aCC-BY 4.0 International license.

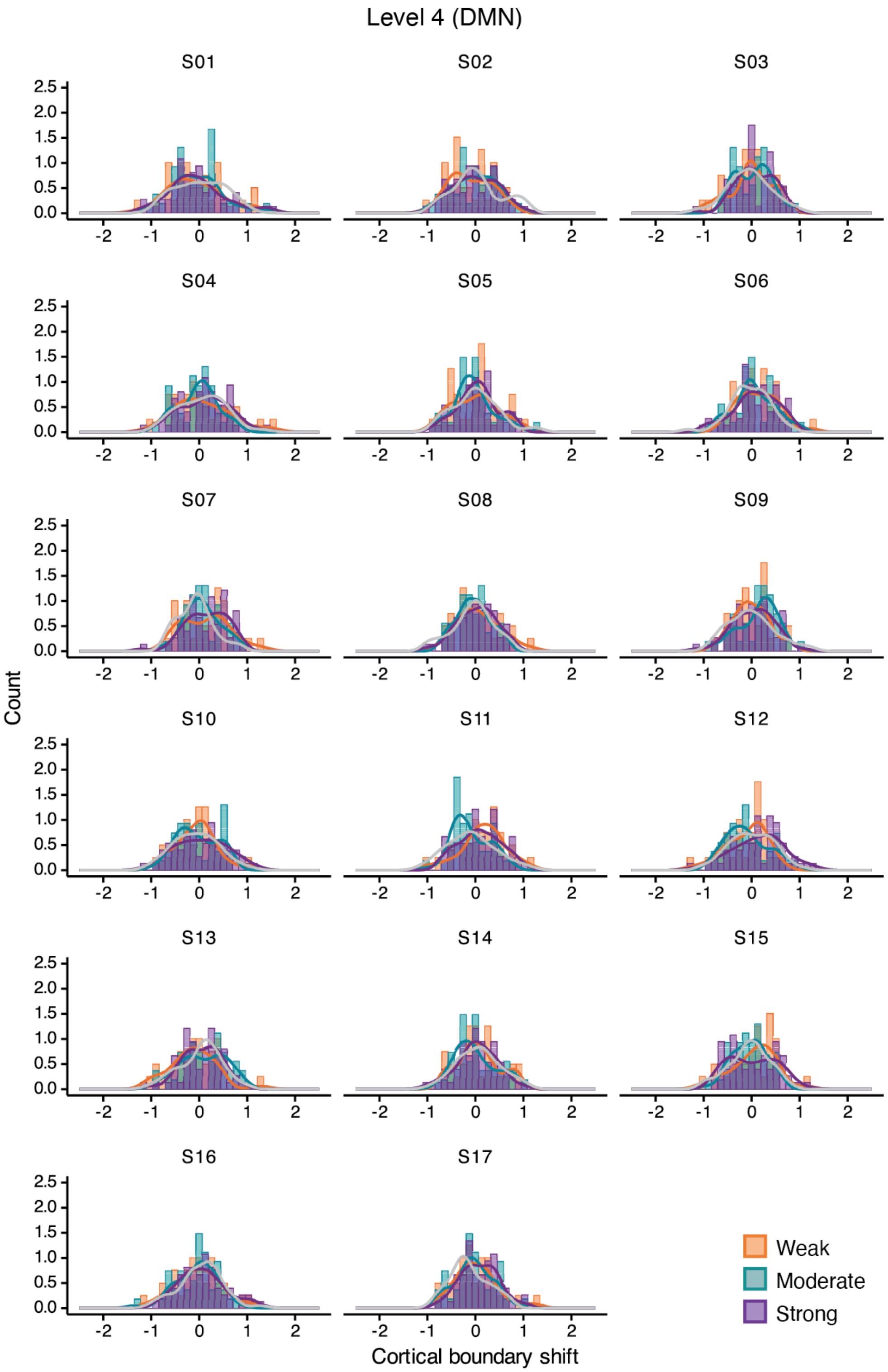

Supplementary Figure 6. Individuals' distributions of cortical boundary shifts for each boundary strength category in the default mode network (DMN) areas at level 4 of the cortical hierarchy. The gray line depicts a density curve of the non-boundary data.

bioRxiv preprint doi: https://doi.org/10.1101/2024.04.10.588931; this version posted June 15, 2024. The copyright holder for this preprint (which was not certified by peer review) is the author/funder, who has granted bioRxiv a license to display the preprint in perpetuity. It is made available under aCC-BY 4.0 International license.

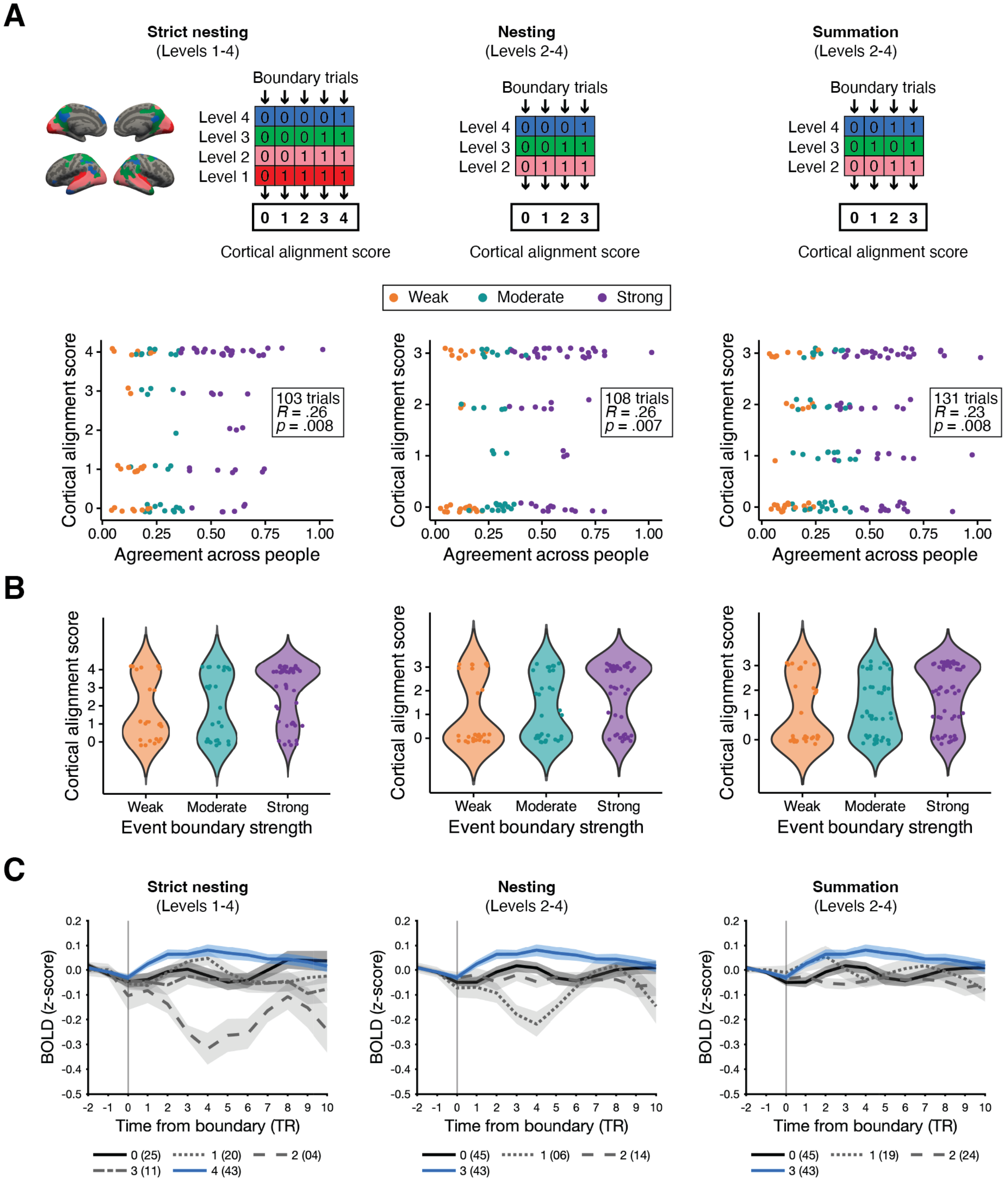

Supplementary Figure 7. Three types of scoring methods (strict nesting, nesting, and summation). Due to the absence of a bottom-up nested structure in cortical pattern shifts, 21.4% and 17.6% of event boundary trials were excluded for strict nesting and nesting, respectively. (A) There was a significant positive relationship between the degree of cortical alignment and boundary agreement across people for all scoring methods (Spearman's rank correlation). In the scatter plot, individual dots depict event boundary trials. (B) The same data are presented in a violin plot to visualize the distribution. (C) Each line illustrates hippocampal activity at event boundaries with different alignment scores for each scoring method (blue solid line: the highest possible alignment score; black solid line: the lowest possible alignment score; shaded area: ± SEM across subjects). Different line types depict different scores (gray dotted line: score 1, gray dashed line: score 2, gray dash-dotted line in strict nesting: score 3). In the legend, the number in a parenthesis shows

bioRxiv preprint doi: https://doi.org/10.1101/2024.04.10.588931; this version posted June 15, 2024. The copyright holder for this preprint (which was not certified by peer review) is the author/funder, who has granted bioRxiv a license to display the preprint in perpetuity. It is made available under aCC-BY 4.0 International license.

the number of event boundary trials associated with that score. Note that different scoring methods produce different numbers of boundary trials for a given score.

Pairwise comparison		$t(16)$	p (corrected)
Strict nesting	Score 0 vs. Score 1	-0.67	1
	Score 0 vs. Score 2	4.14	.008
	Score 0 vs. Score 3	0.63	1
	Score 0 vs. Score 4	-2.23	.405
	Score 1 vs. Score 2	4.27	.006
	Score 1 vs. Score 3	1.10	1
	Score 1 vs. Score 4	-1.73	1
	Score 2 vs. Score 3	-4.65	.003
	Score 2 vs. Score 4	-6.53	< .001
	Score 3 vs. Score 4	-3.19	.057
Nesting	Score 0 vs. Score 1	2.83	.073
	Score 0 vs. Score 2	0.56	1
	Score 0 vs. Score 3	-2.14	.291
	Score 1 vs. Score 2	-3.92	.007
	Score 1 vs. Score 3	-5.01	< .001
	Score 2 vs. Score 3	-3.23	.031

Supplementary Table 3. Post-hoc pairwise comparison (paired t-test) results in the hippocampus analysis. Bonferroni correction was applied. The corrected p-value is the initial p-value multiplied by the number of comparisons. Note that no significant main effect of cortical alignment was observed with the summation method.

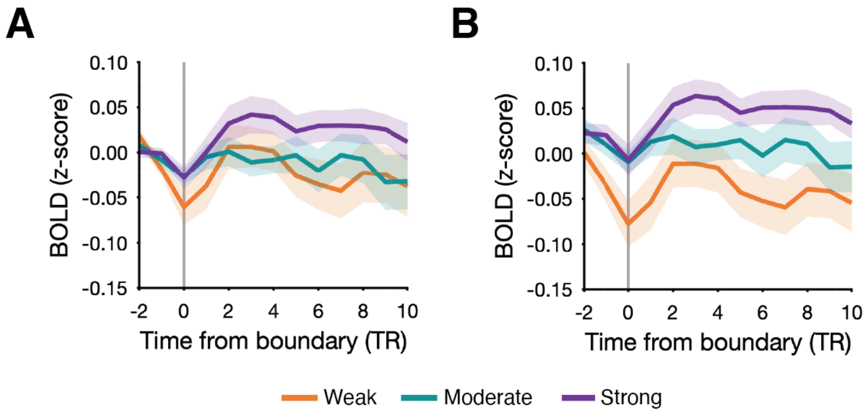

Supplementary Figure 8. Hippocampal response at event boundaries varying with strengths
