# Supplementary material for: The relationship between event boundary strength and pattern shifts across the cortical hierarchy during naturalistic movie-viewing
Source: bioRxiv. 2024 Apr 14:2024.04.10.588931. Preprint. [Version 1] doi: 10.1101/2024.04.10.588931 (PMC11030401; doi:10.1101/2024.04.10.588931)
Supplement: Supplementary file 1 [file NIHPP2024.04.10.588931v1-supplement-1.pdf]

bioRxiv preprint doi: https://doi.org/10.1101/2024.04.10.588931; this version posted April 14, 2024. The copyright holder for this preprint (which was not certified by peer review) is the author/funder, who has granted bioRxiv a license to display the preprint in perpetuity. It is made available under aCC-BY 4.0 International license.

	**Proportion of human observers**	
	**Minimum**	**Maximum**
Weak	.11	.19
Moderate	.19	.40
Strong	.40	.98

Supplementary Table 1. Agreement range for each event boundary strength category.

bioRxiv preprint doi: https://doi.org/10.1101/2024.04.10.588931; this version posted April 14, 2024. The copyright holder for this preprint (which was not certified by peer review) is the author/funder, who has granted bioRxiv a license to display the preprint in perpetuity. It is made available under aCC-BY 4.0 International license.

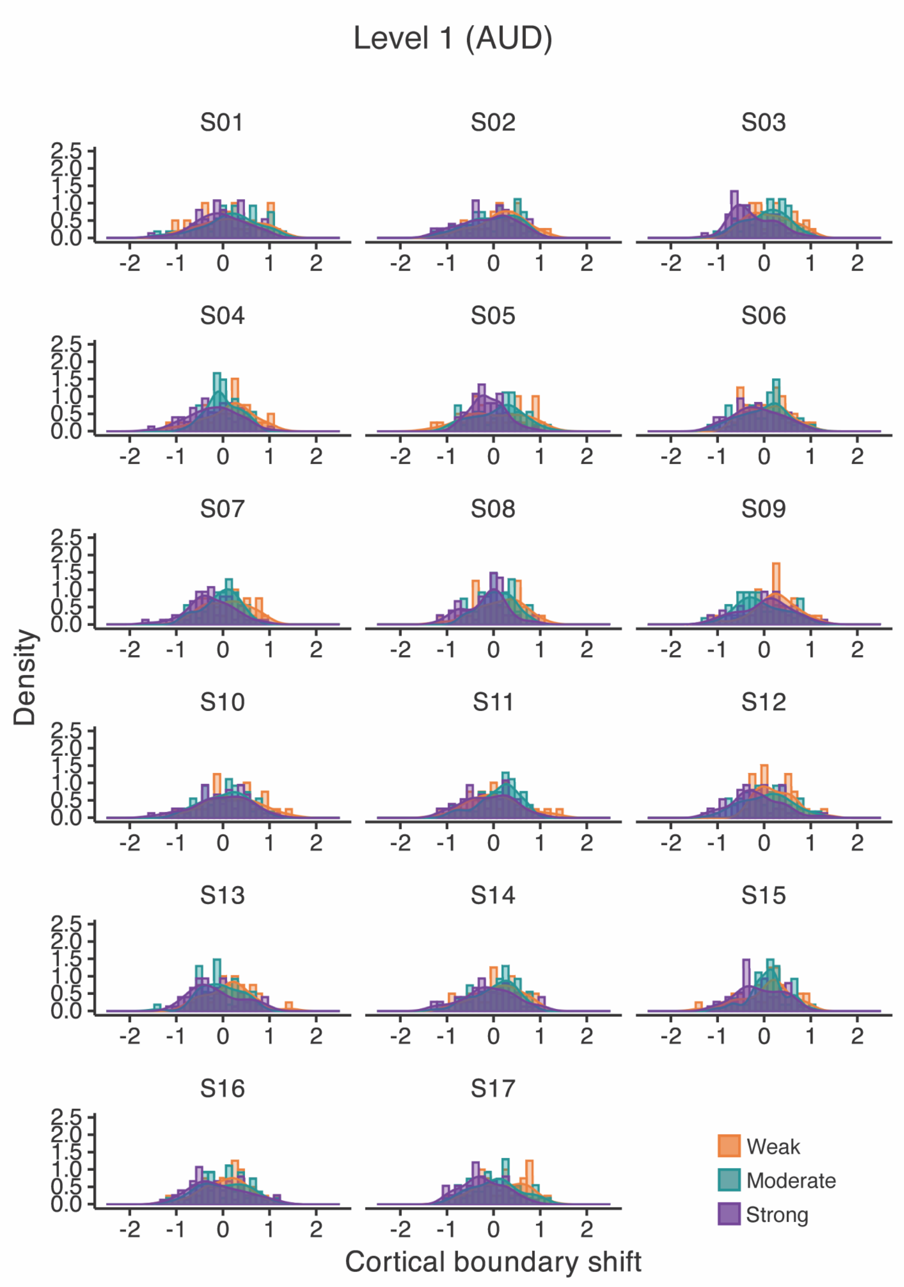

Supplementary Figure 1. Individuals' distributions of cortical boundary shift values for each boundary strength category in auditory processing areas (AUD) at level 1 of the cortical hierarchy.

bioRxiv preprint doi: https://doi.org/10.1101/2024.04.10.588931; this version posted April 14, 2024. The copyright holder for this preprint (which was not certified by peer review) is the author/funder, who has granted bioRxiv a license to display the preprint in perpetuity. It is made available under aCC-BY 4.0 International license.

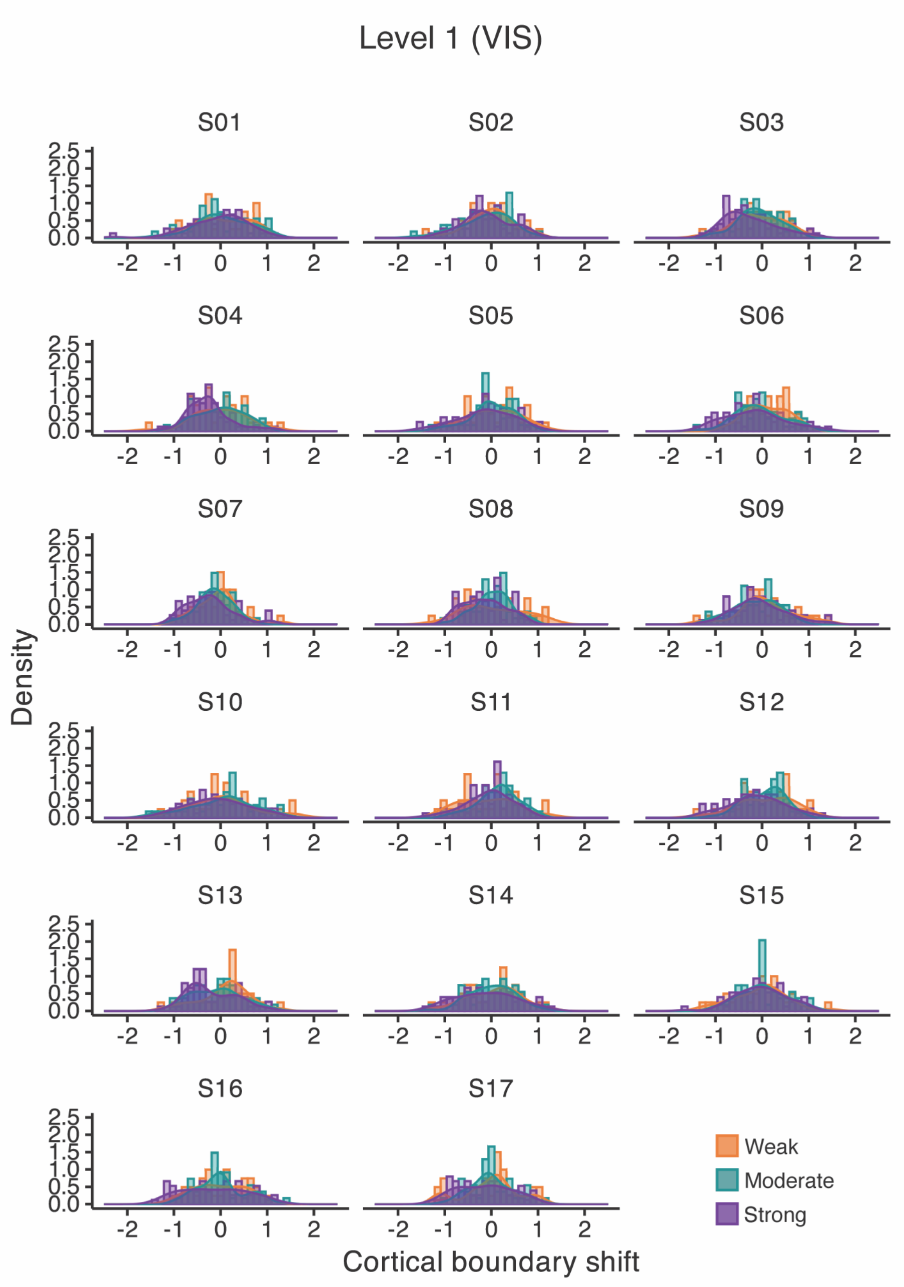

Supplementary Figure 2. Individuals' distributions of cortical boundary shift values for each boundary strength category in visual processing areas (VIS) at level 1 of the cortical hierarchy.

bioRxiv preprint doi: https://doi.org/10.1101/2024.04.10.588931; this version posted April 14, 2024. The copyright holder for this preprint (which was not certified by peer review) is the author/funder, who has granted bioRxiv a license to display the preprint in perpetuity. It is made available under aCC-BY 4.0 International license.

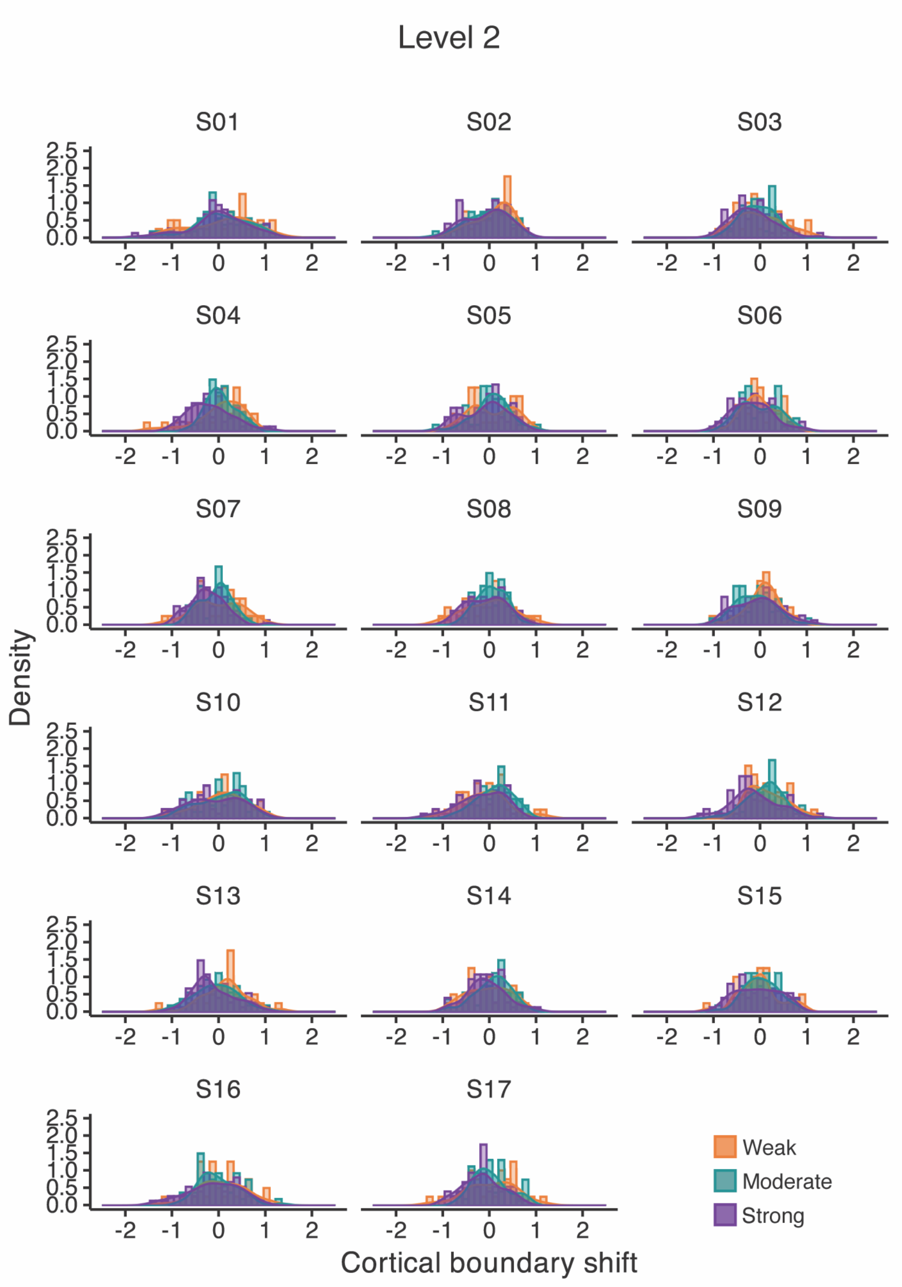

Supplementary Figure 3. Individuals' distributions of cortical boundary shift values for each boundary strength category in the areas at level 2 of the cortical hierarchy.

bioRxiv preprint doi: https://doi.org/10.1101/2024.04.10.588931; this version posted April 14, 2024. The copyright holder for this preprint (which was not certified by peer review) is the author/funder, who has granted bioRxiv a license to display the preprint in perpetuity. It is made available under aCC-BY 4.0 International license.

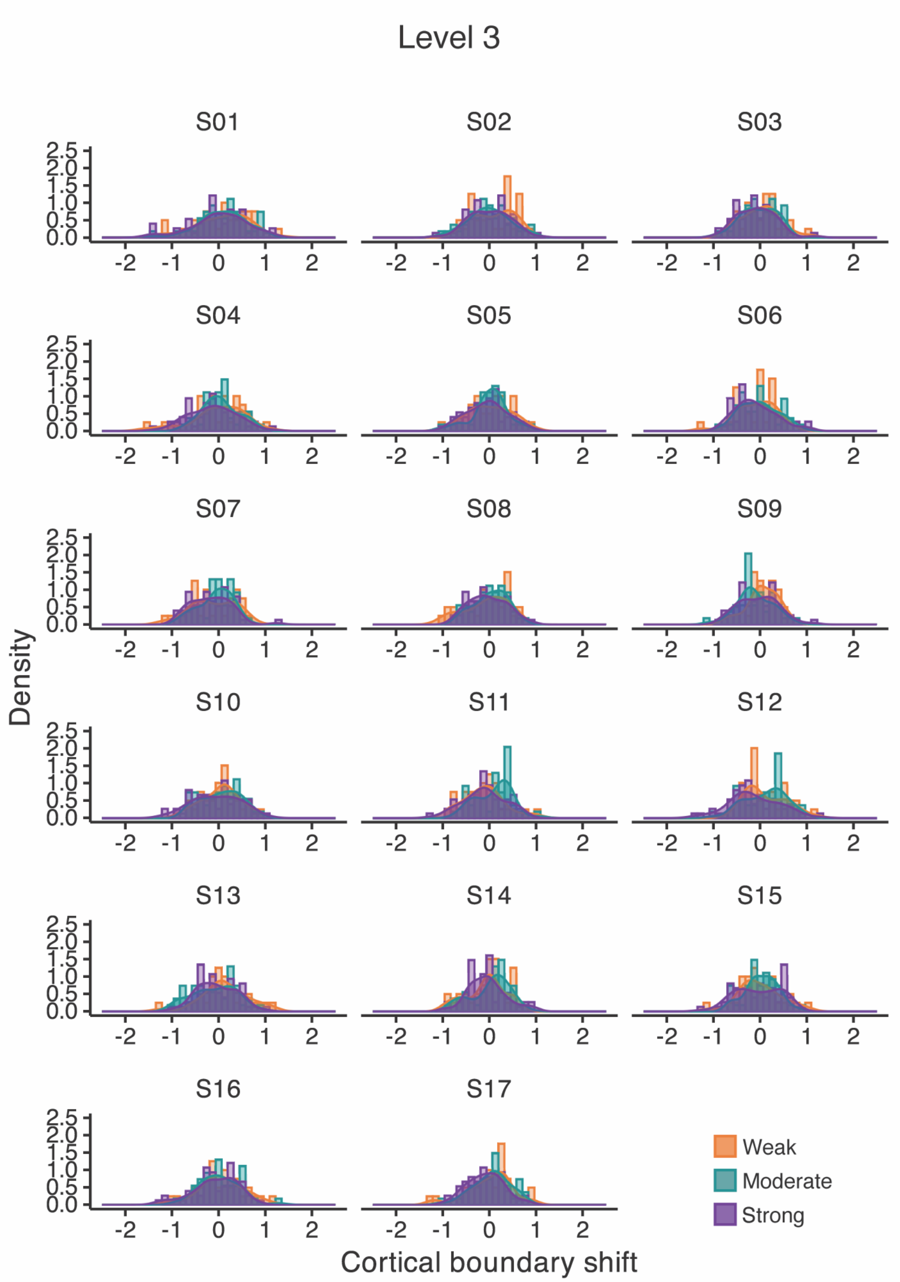

Supplementary Figure 4. Individuals' distributions of cortical boundary shift values for each boundary strength category in the areas at level 3 of the cortical hierarchy.

bioRxiv preprint doi: https://doi.org/10.1101/2024.04.10.588931; this version posted April 14, 2024. The copyright holder for this preprint (which was not certified by peer review) is the author/funder, who has granted bioRxiv a license to display the preprint in perpetuity. It is made available under aCC-BY 4.0 International license.

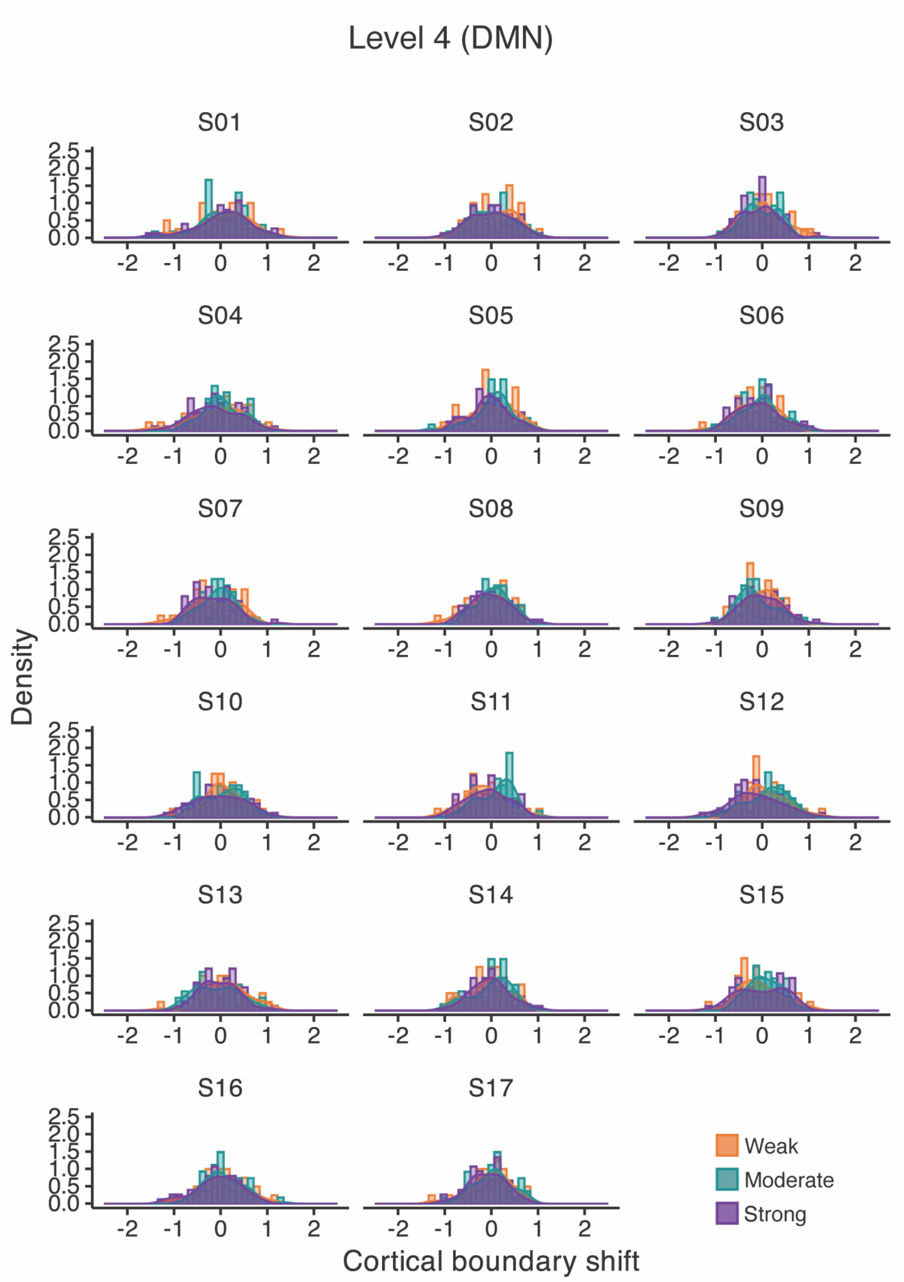

Supplementary Figure 5. Individuals' distributions of cortical boundary shift values for each boundary strength category in the default mode network (DMN) areas at level 4 of the cortical hierarchy.
